# *Bacillus amyloliquifaciens*-Supplemented Camel Milk Suppresses Neuroinflammation of Autoimmune Encephalomyelitis in a Mouse Model by Regulating Inflammatory Markers

**DOI:** 10.3390/nu15030550

**Published:** 2023-01-20

**Authors:** Hairul Islam Mohamed Ibrahim, Abdullah Sheikh, Hany Ezzat Khalil, Ashraf Khalifa

**Affiliations:** 1 Biological Science Department, College of Science, King Faisal University, P.O. Box 400, Al-Ahsa 31982, Saudi Arabia; 2 Molecular Biology Division, Pondicherry Centre for Biological Sciences and Educational Trust, Kottakuppam 605104, India; 3 Camel Research Center, King Faisal University, P.O. Box 400, Al-Ahsa 31982, Saudi Arabia; 4 Department of Pharmaceutical Sciences, College of Clinical Pharmacy, King Faisal University, Al-Ahsa 31982, Saudi Arabia; 5 Department of Pharmacognosy, Faculty of Pharmacy, Minia University, Minia 61519, Egypt; 6 Botany and Microbiology Department, Faculty of Science, Beni-Suef University, Beni-Suef 62511, Egypt

**Keywords:** *Bacillus amyloliquifaciens*, fermented camel’s milk, multiple sclerosis, probiotics, VCAM signals

## Abstract

Multiple sclerosis (MS), a distinct autoimmune neuroinflammatory disorder, affects millions of people worldwide, including Saudi Arabia. Changes in the gut microbiome are linked to the development of neuroinflammation via mechanisms that are not fully understood. Prebiotics and probiotics in camel milk that has been fermented have a variety of health benefits. In this study, *Bacillus amyloliquefaciens*-supplemented camel milk (BASY) was used to assess its preventive effect on MS symptoms in a myelin oligodendrocyte glycoprotein (MOG)-immunized C57BL6J mice model. To this end, MOG-induced experimental autoimmune encephalomyelitis (EAE) was established and the level of disease index, pathological scores, and anti-inflammatory markers of BASY-treated mice using macroscopic and microscopic examinations, qPCR and immunoblot were investigated. The results demonstrate that BASY significantly reduced the EAE disease index, increased total microbial load (2.5 fold), and improved the levels of the short-chain fatty acids propionic, butyric and caproic acids in the diseased mice group. Additionally, myeloperoxidase (MPO) proinflammatory cytokines (IL-1β, IL-6, IL-17, TNF-α) and anti-inflammatory cytokines (TGF-β) were regulated by BASY treatment. Significant suppression of MPO and VCAM levels were noticed in the BASY-treated group (from 168 to 111 µM and from 34 to 27 pg/mL, respectively), in comparison to the EAE group. BASY treatment significantly reduced the expression of inflammatory cytokines, inflammatory progression related transcripts, and inflammatory progression protein markers. In conclusion, BASY significantly reduced the symptoms of EAE mice and may be used to develop a probiotic-based diet to promote host gut health. The cumulative findings of this study confirm the significant neuroprotection of BASY in the MOG-induced mice model. They could also suggest a novel approach to the treatment of MS-associated disorders.

## 1. Introduction

Multiple sclerosis (MS) is a distinct autoimmune neurological disorder accompanied by inflammatory conditions that affects over 2.8 million people worldwide, including Saudi Arabia [1,2,3]. The exact pathological mechanisms underlying the development of MS are not yet fully understood. Nevertheless, the cascade of MS pathology comprises demyelination, oligodendrocyte loss, neuronal loss, axonal damage, and progressive failure of remyelination [4,5,6]. Studies indicate that axonal injury plays a key role in the persistence of neurological deficits, and unambiguously, demyelination is considered the pathologic hallmark of MS [7]. Reports proposed that inflammatory conditions could attack and degenerate brain axons and neurons in people with MS and lead to disabilities [8,9,10]. The implementation of anti-inflammatory and immunomodulatory management plans can therefore prevent or even delay the progression of MS.

Recent evidence suggests that the modulation of the gut microbiome (the GM) could moderate the risk of MS, an autoimmune disease that is the leading cause of non-traumatic neurological disability, but the interaction of bacterial species and the host genetics has not yet been explored [11]. The GM is part of a complex ecosystem that holds great potential and plays a crucial role in the regulation of physiological processes such as metabolism and immunity. The GM can also affect brain functions through interactions with the immune, nervous and endocrine systems [12,13]. Mechanistically, the GM can produce metabolites that have the potential to modulate inflammation via their interactions with the central nervous system. Studies have revealed a relationship between the intestinal mucosa and the brain via the production of polysaccharide A by *Bacillus fragilis* [14]. Moreover, the GM can affect the release of cytokines. Consequently, the GM may regulate the release of neurotransmitters in both the central and peripheral nervous systems. This kind of unique regulation due to interactions between the GM and the host can lead to the production of biogenic amines and neuroactive mediators, as well as the expression of an immune-regulatory role by host cells [15]. These distinctive interactions can affect important processes within the body, including the immune and nervous systems, thereby aiding the treatment of MS [16]. Furthermore, the GM plays an important role in the fermentation process, in particular in the conversion of indigestible carbohydrates into short-chain fatty acids (SCFAs), which are reputed to decrease inflammation and contribute to host immunity [17,18]. Studies using the animal model of MS and experimental autoimmune encephalomyelitis (EAE) have reported strong evidence suggesting a link between the GM and MS development [19]. The GM can directly interact with the immune system through numerous mechanisms, including modulation of the host microRNAs (miRNAs) affecting gene expression at the post-transcriptional level, or production of microbial metabolites [20,21]. Various miRNAs (for example, miR-141 and miR-200a) that mediate host–microbe interactions leading to the shaping of the microbiome structure and mediating inflammatory and autoimmune diseases have been studied [20,21].

Probiotic bacteria are typically grown in a lab or food production facility. They are administered orally in adequate daily doses to promote health via restoration of the GM and reduction of intestinal inflammation and oxidative stress, and an improved immune system [22]. It is reported that the most common bacteria used as probiotics are *Lactobacilli, Bifidobacteria*, *Enterococci* and *Bacillus* [23]. Preclinical studies have shown that probiotic consumption reduces the incidence and severity of MS [24]. Numerous studies demonstrated the ameliorative effects of probiotics on the host as functional food via the normalization of the imbalanced GM [1]. Furthermore, the use of probiotics improved immune/inflammatory processes in some diseases, such as diabetes [25], inflammatory bowel disease [26], neuro-inflammatory disorders and MS [27]. The neurotransmitter γ aminobutyric acid (GABA) has been found to maintain intracellular redox homeostasis and protect neurons from oxidative damage [28]. Moreover, further evidence showed the level of GABA was reduced in neuropsychiatric diseases [29,30]. Interestingly, intestinal microbiota, such as *Lactobacillus* SPP and *Bifidobacterium*, can produce GABA mediators [31]. It was also evidenced that treatment with probiotics for 4 weeks significantly improved the gastrointestinal symptoms in patients with Parkinson’s disease through the secretion of GABA factors.

Camel milk (CM) is endowed with bio-functionalities—including antioxidant, anti-inflammatory and controlling of inflammatory bowel syndrome (IBS)—by maintaining the proper functioning of the intestinal barrier and by reducing proinflammatory cytokines in gut tissue [32]. Reports evidenced that the administration of fresh CM mitigated the colonic levels of proinflammatory and necrotic cytokines [33]. CM has a distinctive composition and contains vital substrates on which fermenting bacteria can readily grow, producing many bio-functional constituents in the process [34]. One of the studies reported that the gut microbiota influenced brain function and behavior through neurological, immunological, and endocrine pathways, establishing the microbiota–gut–brain axis (MGBA) [35]. The fermented CM contained various bioactive metabolites, such as antioxidative peptides, which were absent from the fresh (unfermented) CM [36]. Based on current strategies for the importance of functional-fermented foods, fermented CM could serve as a potential source of bioactive nutrients for the management of various diseases [32]. Our previous work highlighted the modulatory effects of CM enriched with *Bacillus amyloliquefaciens* (BA) on proinflammatory cytokines including interleukin-1β (IL1β), interleukin 6 (IL6), interleukin 8 (IL8), and tumor necrosis factor alpha (TNF-α) in induced IBS in a mice model [28]. It is thus of interest to study CM fortified with BA, as an example of a probiotic that could offer a complementary approach for the management of MS. Specifically, it would be beneficial to explore how BA-based probiotics and dietary modifications of CM could be used to mitigate the effects of MS based on their effect on the GM, which play a pivotal role in the pathophysiology of MS. This approach could be applied to develop microbiome-based therapies for the treatment of MS.

In this study, BA-supplemented camel milk (BASY) was used to test the ability of the MOG-immunized C57BL6J mouse model to prevent MS symptoms. To this end, myelin oligodendrocyte glycoprotein (MOG)-induced experimental autoimmune encephalomyelitis (EAE) was generated, and the disease index, pathological scores, and inflammatory and anti-inflammatory markers of BASY-treated mice using macroscopic and microscopic examinations, qPCR and immunoblot were investigated.

## 2. Materials and Methods

### 2.1. Bacterial Strain and Preparation of Yogurt

The probiotic bacterial strain (BA (JF836079)) used in this study was previously characterized for its anti-oxidative and immunomodulatory traits. The BA was routinely maintained at −80 °C in De Man, Rogosa, and Sharpe agar cryostocks, and was regularly sub-cultured in MRS broth for 18 h at 37 °C before use. Camel milk was gently mixed and inoculated with BA under aseptic conditions after being pasteurized at 68 °C for 20 min and cooled to 42 °C in a cold water bath [37]. A natural camel milk based yogurt (control without BA) and a yogurt enriched with BA (inoculated with 10^8^ CFU/g of the BA) were the two groups of yogurt preparations used in the study. When the pH reached 4.5 ± 0.05, the yogurt was aseptically poured into sterilized polystyrene cups and incubated at 40 °C for approximately 5 h. The yogurt was then immediately chilled and kept at 4 °C for 24 h, where it could mature in the cold and be used later.

### 2.2. Animal Care and Experimental Design for EAE Induction and Clinical Evaluation

Male C57Bl6j mice aged six weeks and weighing between 18 and 22 g were used for the experiments; they were purchased from King Faisal University’s Animal House (KFU). All animal testing was done in accordance with the deanship of scientific research ethical committee’s code of ethics for the treatment and use of laboratory animals (KFU-REC-2021-OCT-EA00075). Mice were immunized with 100 mg of myelin oligodendrocyte glycoprotein peptide 35-55 (MOG35-55) emulsified in complete Freund’s adjuvant (CFA) supplemented with 5 mg/mL of *Mycobacterium tuberculosis* H37Ra to induce classical active EAE. Four locations on the mice’s flanks received subcutaneous injections of 200 µL of emulsion altogether. The animals received additional intravenous injections of 100 ng pertussis toxin (PTX) at days 0 and 2 following the initial peptide injections. Naive CD4+ T cells from C57BL6j mice were polarized in vitro into Th17 or Th1 cells as previously described [38] in order to induce EAE through adoptive transfer. The following score system was used to evaluate the common EAE symptoms: scores range from 0 (no disease) to 5 (moribund or dead)—with 0 meaning no disease; 0.5 meaning reduced tail tonus; 1 meaning a limp tail; 1.5 meaning impaired righting reflex; 2 meaning a limp tail and weakness in the hind limbs; 2.5 meaning at least one hind limb is paralyzed; 3 meaning both hind limbs are paralyzed; 3.5 meaning complete paralysis of the hind limbs; and 4 meaning paralysis until the hip. The following score system was used to assess the atypical EAE symptoms: score 0 indicates no disease; score 1 indicates a slight head turn (ataxia, no tail paralysis); score 2 indicates a more pronounced head turn; score 3 indicates an inability to walk straight; score 4 indicates the animal lying on its side; score 4.5 indicates a continuous rolling motion unless supported; and score 5 indicates moribund or death. The highest score from the typical or atypical clinical signs is taken into account in the final score combining typical and atypical scoring [39]. If a mouse’s score rose above 3, they were put to death.

### 2.3. Antibiotic Treatment

The mice were grouped, and microbial clearance from ileal content was done using 2.5 mg/mL norfloxacin in drinking water, followed by 0.8 mg/mL of amoxicillin, and 0.114 mg/mL clavulanic acid in drinking water for one week [40]. After one week of antibiotic treatment, BASY was administered for 25 days and followed with MOG/CFA/PTX induction for EAE disease developments.

### 2.4. Classification of Paralysis Symptoms

Daily weight and paralysis signs were monitored in the EAE mice. The following key was used to score the symptoms of paralysis: no symptoms (0), inability to curl the distal end of the tail (1), complete tail atony (2), impaired movement (3), partial hind limb paralysis (4), complete hind limb paralysis (5), and tetraplegia (6).

### 2.5. Isolation of Cells

Neural tissue dissociation kits from Miltenyi (Miltenyi Biotec, Charlestown, MA, USA) were used to create single cell suspensions in accordance with the manufacturer’s instructions [41]. Myelin was removed from the single-cell suspensions using two washes in 30% percoll following the dissociation of CNS tissue. The remaining cells were then suspended in RPMI supplemented with FBS and penicillin/streptomycin (cRPMI) for later use after being subjected to RBC lysis buffer, washing, and cRPMI. Mechanical dissociation was used to separate the immune cells from the spleens and lymph nodes, and then the cells were RBC-lysed and filtered through 70 µm filters. The remaining mononuclear fractions were then suspended in cRPMI for use downstream after washing.

### 2.6. ELISA Quantification of Secreted Cytokines

For the ELISA quantification of interleukin 1β, interleukin 6 (IL-6) and interleukin 17 (IL-17), cell isolation media samples were collected from primary cultures of mononuclear cells isolated from the CNS and spleen and plated at 10 10^6^ cells/mL under unstimulated conditions from EAE mice treated in vivo, and under MOG35-55 (20 g/mL) stimulated conditions from MOG35-55-immunized mice treated in vitro. ELISAs were carried out in accordance with Biolegend’s instructions [42]. By comparing the relative absorbance of variable samples at a wavelength of 450 nm to the standard curve calculated from standards of known concentration, the concentration of captured protein content was determined. A Biotek multiplate reader was used to analyze the cytokine plates.

### 2.7. In Vitro Stimulation of MOG35-55 Reactive Mononuclear Cells

C57BL/6 mice were vaccinated with 150 g of MOG35-55 and 600 mg of H37Ra suspended in an emulsion of sterile PBS and Freund’s adjuvant. The mice were put to death with isoflurane after 7 days. In order to isolate the cells, the mice’s spleens were removed and subjected to mechanical dissociation, RBC lysis, and 70 m filtration. In complete cRPMI activated with 20 µg/mL MOG35-55, live cells were plated at a density of 10^6^ cells per mL. Activated cells were cultured for 48 h with or without 100 µM tryptamine in the medium.

### 2.8. Histological Analysis

Prior to being put to sleep, mice were perfused with 10 mL of 10% formalin and 10 mL of heparinized PBS. Spinal cords were then preserved in 70% EtOH and set in paraffin blocks after being isolated. Using a water bath and a microtome, 4 µm sections were cut and adhered to microscope slides. According to previously published protocols, slides were deparaffinized with xylene and ethanol washes before being stained with the hematoxylin and eosin (H&E) [43].

### 2.9. Markers of Inflammation in the Spinal Cord and Colon Tissues

The quantities of cytokines (granulocyte-macrophage colony-stimulating factor (GM-CSF), interleukin-1 beta (IL-1β), interleukin 6 (IL-6), IL-17 and transforming growth factor β (TGF-β)) were determined in the spinal cord and colon tissues of MOG-immunized EAE mice using ELISA kits (Invitrogen, Thermo Fisher Scientific, Vienna, Austria; Cayman, CA, USA) following the instructions of the manufacturers. The levels of cytokines on the plates were reported at 450 nm on an automated ELISA plate reader (BioTek Instruments, Vermont, United States).

### 2.10. Myeloperoxidase (MPO) Activity

The activity of MPO, a marker of neutrophilic infiltration, was determined in the spinal cord and distal colon, according to the method described earlier [42]. Briefly, the spinal cord and colon (~1 cm length) was obtained and homogenized (50 mg/mL) in ice-cold 50 mM PBS (pH 6) containing 5% hexadecyl trimethyl ammonium bromide (Sigma-Aldrich, St. Louis, MO, USA). It was incubated at 90 °C for 30 min, and briefly centrifuged to remove the residues. The absorbance was measured for the colorimetric reaction using a spectrophotometer (Thermo Scientific, Waltham, MA, USA). MPO activity is expressed as units per milligram of wet tissue. One unit expresses the MPO activity needed for the conversion of 1 mM of H_2_O_2_ to water in 1 min at room temperature.

### 2.11. Quantification of SCFAs

Using the previously described methods, the content of SCFA was calculated from the cecal content [44]. Briefly, a mixture of 400 µL of deionized water and 100 mg of cecal content was homogenized. Samples were homogenized using phosphoric acid, left for 30 min and then centrifuged (12,000× *g* for 15 min). The internal standard used to precisely determine the experimental concentrations was ethyl butyric acid (Sigma, Sigma Aldrich, St. Louis, MO, USA). Capric acid (Himedia, Mumbai, India) was added to each sample to a final concentration of 0.0375 mM. Samples were analyzed using BIOvision chemical kits.

### 2.12. Quantitative PCR Evaluation and RNA Isolation

Utilizing the RNeasy Mini Kit and following the manufacturer’s instructions, RNA was extracted from tissue samples. Equivalent amounts of RNA were used to create cDNA using the Superscript II RT (Invitrogen, Carlsbad, CA, USA), and the PowerUp SYBR Green Master Mix was used to amplify the PCR products. On the StepOne real-time PCR system, samples were normalized using b-actin, and relative mRNA expression was measured using the comparative CT method. The expression of specific gene transcripts was measured by using the primer pairs presented in Table 1. A melting point analysis was carried out to improve the sensitivity and specificity of amplification reactions detected with the SYBR green I dye. Data were analyzed by quant studio Software (Applied Biosystems, Foster City, CA, USA) following the 2^−ΔΔCT^ method [45]. The significance of differences was assessed using the Student’s *t*-test.

### 2.13. Western Blot

Using RIPA lysis buffer, the spinal cord and distal colon of EAE mice and BASY were used to produce the protein lysate (Santa Cruz, CA, USA). Following separation on an SDS-PAGE gel (10%), the spinal lysates were transferred to a PVDF 0.22 µM membrane. Transferred blots were incubated overnight with primary antibodies at 4 °C with respective antibodies such as MBP (mouse monoclonal antibody 1:1500) (Invitrogen, Waltham, MA, USA), VEGF (rabbit polyclonal antibody 1:1000) (Invitrogen, Waltham, MA, USA), GABA (rabbit polyclonal antibody 1:1000) (Biorbyt, Cambridge, UK), COX2 (rabbit polyclonal antibody 1:1000), BDNF (rabbit monoclonal antibody 1:1000) (Biorbyt, Cambridge, UK), and Gapdh (rabbit polyclonal antibody 1:1500) (Biorbyt, Cambridge, UK). The quantifications were carried out using the densitometry tool in ImageJ software version 1.8 after checking the chemiluminescence (Licor signal, MA, USA) of the expressed bands to confirm the linear range of the chemiluminescence signals.

### 2.14. Statistical Analysis

Version 8.4.3 of the GraphPad Prism program was used for the statistical analysis. To compare experimental groups divided by a single variable, unpaired t-tests were applied. To compare changes in experimental groups that had a single subject in common before being exposed to a variable, paired t-tests were used. A solid line connecting individual values between groups annotates common topics. We used four experiments with five mice per group, totaling 20 mice, to test the effectiveness of tryptamine in EAE. The legends of the figures show how many animals were used in other experiments. Every graph displays a single value along with the mean and standard error of the mean (SEM). Samples were considered statistically significant if *p* < 0.05. The degree of significance was demonstrated using the following key: * *p* < 0.05, ** *p* < 0.01, *** *p* < 0.001.

## 3. Results

### 3.1. Effect of Immunomodulation and Biocompatibility of BA on HT-29 Colon Cell Lines

BA supplemented food was previously confirmed for its biocompatibility and immunomodulatory effects on HT-29 colon cell lines [32]. The nutritional content of BASY is presented in Table 2. As the BA load in the inoculum increased, the total fat, cholesterol, and total protein increased. However, the total carbohydrate decreased from 4.4 g at BA 10 × 6 to 3.5 at BA 10 × 9 inoculum (Table 2). No significant change was observed for the sodium content, regardless of the bacterial load in the inoculum.

### 3.2. Pathology of EAE C57Bl6j Mice Affected by BASY

**Figure 1:**
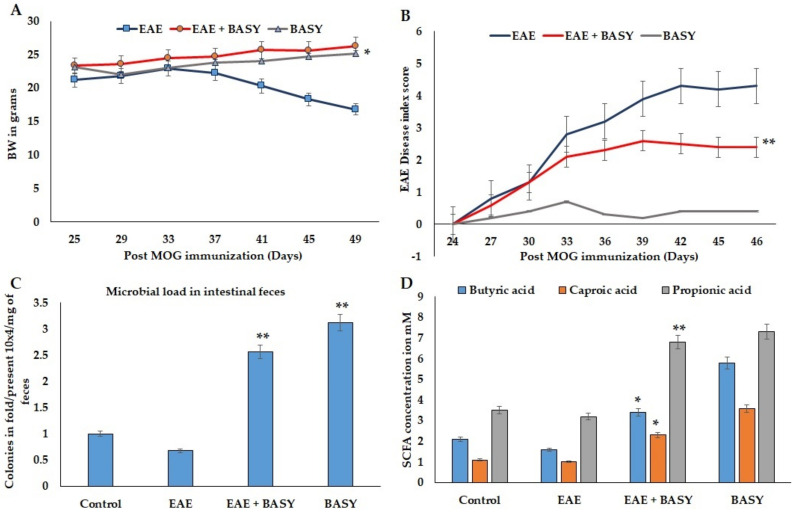
BASY effectiveness on different parameters of MOG-induced EAE mice. The mice were immunized with MOG 35-33 (emulsified with CFA using a T connector). BASY induced the tolerance against EAE induction in mice: (**A**) Body weight (BW) of mice (EAE, EAE+BASY and BASY-treated mice). (**B**) Clinical symptoms recorded post-MOG immunization resulted in gradual increase in paralysis and hind limb inactivity, noted from 11 days of MOG immunization, which became stable after the 18th day post immunization. (**C**) The total microbial load in pre-administered BASY for 25 days of pre-immunization and MOG-immunized mice after the 16th day. The total bacterial count in fecal samples before and after BASY treatment (Group 1: untreated control group; Group 2: MOG-immunized mice (EAE); Group 3: BASY treated with MOG immunization (EAE+BASY); Group 4: BASY–only treated mice). The uncharacterized colonies were counted using the colony counter and recorded as 10^4^ colonies/gram of fecal samples. (**D**) SCFAs, butyric acid, caproic acid, and propionic acid were quantified using Biovision biochemical kits in the ileal content. The values are measured using a microplate reader and expressed as mM/mg of ileal content. Data were pooled from three independent experiments and shown as mean ± SD. * *p* < 0.05, ** *p* < 0.01. (**A**) microbial load compared using a student *t*-test. (**B**) SCFA content was compared using a one-way ANOVA.

The primary modification in the multiple sclerosis model is physiological symptoms. Mice immunized with MOG showed macroscopic changes in their clinical score and disease index. In this study, an in vivo model was used to assess the protective effects of BASY administration on the demyelination and paralysis symptoms of MS mice. At 12 days following MOG immunization, the naive EAE group exhibited MS clinical signs, including hind limb and tail paralysis. Such symptoms gradually developed, peaked at 14 days after MOG immunization, and then persisted (Figure 1A,B). Treatment of EAE mice with BASY significantly decreased the disease impact, and delayed paralysis symptoms for up to 24 days after immunization. BASY ameliorated the disease severity in particular at the hind limb, and with respect to tail paralysis. The total microbial load was significantly increased in the EAE-BASY group, with a 2.5-fold higher level than that of the diseased group (Figure 1C). Three SCFAs were quantified, and the results are presented (Figure 1D). In decreasing order, propionic, butyric, and caproic acids were significantly higher in the BSAY-EAE-treated group than those in the EAE group. These findings indicated that BASY could alleviate EAE symptoms via an increment in microbial load and activation of SCFA. This indicated that BASY displayed potential preventive benefits for mice with EAE induced by MOG.

**Figure 2:**
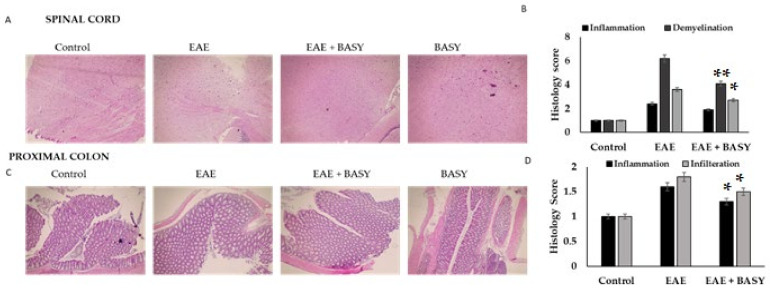
BASY ameliorated the histopathological hallmarks in the CNS and colon of EAE mice. On the 24th day of MOG post-immunization, spinal cords were euthanized from EAE mice, fixed in 10% formaldehyde following paraffin embedding and sectioned into 4 µm thick sections. (**A**) Demyelination was assessed in the spinal cords using H&E, and the pathological scores were recorded at 200× magnification power. (**B**) Infiltration and inflammation scores were noted. (**C**) Intestinal integrity and mucin damage were assessed using H&E of the proximal colon of EAE mice. (**D**) Infiltration of T-lymphoid cells, neutrophils, and inflammation scores were noted on the 24th day of MOG post immunization. Data were pooled from three independent experiments and shown as mean ± SD. * *p* < 0.05. ** *p* < 0.01.

BASY treatment substantially improved motor skills and ameliorated the demyelination of the spinal cords of EAE mice. The investigation of the histological signs of the spinal cord white matter, the percentage of demyelination (~50% recovery) and the T cell inflammatory infiltration density (~33% reduction) were significantly reduced by BASY treatment of EAE mice (Figure 2A). Additionally, the level of inflammatory damage was significantly reduced by 72% in EAE mice treated with BASY. Simultaneously, the pathophysiology score of the proximal colon of the BASY-treated mice showed a reduction in neutrophil infiltration and endothelial integrity (Figure 2C). The histology score of the inflammation and infiltration in the EAE mice showed 1.6 and 1.8 fold increases, respectively; whereas the BASY preadministered mice showed 1.3 and 1.4 fold reductions, respectively.

### 3.3. Effect of BASY on Inflammatory Markers in MOG-Induced EAE Mice

**Figure 3:**
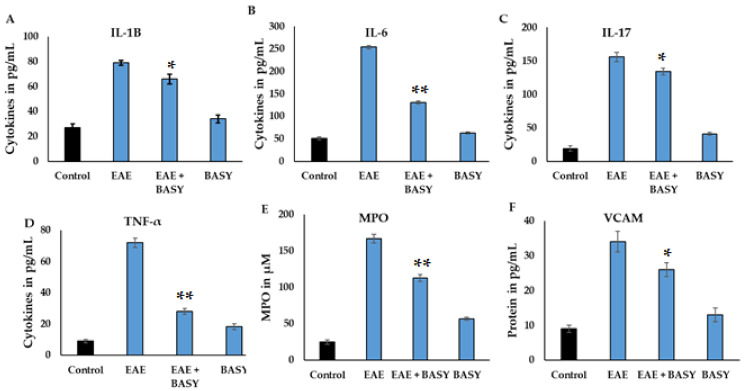
Effect of BASY on inflammatory markers (IL-1B, IL-6, IL-17 and TNF-α), oxidative damage (MPO) and cell adhesion (VCAM) markers in CNS of MOG-induced EAE mice. These markers were quantified in MOG-induced EAE mice and BASY-treated mice after 24 days of the experiment. The protein markers were extracted from the CNS of MOG-immunized mice. The tissues were homogenized after 21 days of induction using RIPA lysis buffer. The cytokines and chemokines were quantified using Invitrogen and Cayman ELISA kits. (**A**) IL-1B was quantified using an Invitrogen kit. The TMB optical variation was quantified at 450 nm by the microplate reader, and values were expressed in pg/mL. (**B**–**D**) IL-6, IL-17 and TNF-alpha were quantified using the Cayman kit. (**E**,**F**) The oxidative stress marker MPO and adhesion molecule VCAM were quantified in the periphery of the spinal cord tissues, respectively. All data were collected from three individual experiments, pooled, and expressed as mean ± SD (*p* < 0.05). * *p* < 0.05 and ** *p* < 0.01 represents significance compared to the MOG vs. MOG+ BASY group. IL—interleukin; TNF-α—tumor necrosis factor alpha; MPO—myeloperoxidase; VCAM—vascular cell adhesion molecule.

The effects of BASY on the four inflammatory markers namely IL-1B, IL-6, IL-17 and TNF-α, in MOG-induced EAE mice were evaluated (Figure 3A–D). The results displayed considerable changes in activity of these cytokines between the treated and control groups in EAE models (Figure 3A–D). The IL-1B, IL-6, IL-17 and TNF-α were significantly increased in the EAE group (78.2 ± 2.5pg/mL, 246.5 ± 10.1, 151 ± 7.2, 72 ±3.5 pg/mL) compared to the BASY-preadministered EAE group (63.1 ± 4.3, 110 ± 7.3, 128.5 ± 11.2, 26.1 ± 3.2 pg/mg, respectively). Significant suppression of MPO and VCAM levels were noticed in the BASY-treated group (from 168 to 111 µM) and (from 34 to 27 pg/mL), respectively (*p*< 0.05), in comparison to the EAE group. Overall, the BASY-treated MOG-induced group showed a remarkable reduction (*p*< 0.05) in TNF-α activity among all of the treatment groups. These cytokine patterns displayed the substantial suppression of T cell polarization and proinflammatory cytokines. It is evident that BASY exhibited a profound modulatory effect on the release of inflammatory cytokines in the CNS of EAE mice.

### 3.4. BASY Activated Treg Cells and Alleviated EAE Symptoms

**Figure 4:**
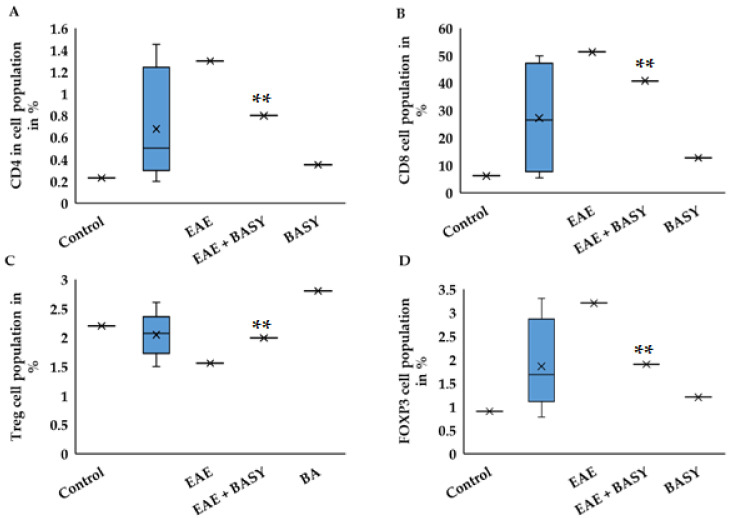
BASY activates the Treg cell population and the EAE condition by controlling CD4 polarization. (**A**–**D**) absolute number of infiltratedCD4, CD8, Treg and FOXP3 positive cells within the CNS of the control, EAE, EAE+BASY, and BASY pretreated MOG-immunized mice groups, respectively. The cells were sorted using the Miltenyi cell separation kit and the total population was counted using the Thermos cell counter. Incidence bars in each graph are expressed as the upper and lower limit of cell population in CNS of EAE and BASY with EAE mice groups. Data were pooled from three independent experiments and shown as mean ± SD. ** *p* < 0.01.

The induced immunoregulatory signals, which are involved in inflammation, alleviated clinical signs in BASY preadministered EAE mice. As can be seen in Figure 4A–D, BASY substantially decreased the CD4, CD8 and FOXP3 populations of Group 3, whereas the Treg cell population was significantly increased compared with the EAE-diseased group. The polarization of Treg cell increased from 1.5 to 1.9 × 10^4^ in CNS of MOG-immunized mice treated with the BASY group, which could be related to clinical improvement (Figure 2A). However, no significant difference was observed in the CD8 cell population, indicating well-balanced immunoregulatory effects of the BASY treatment (Figure 4B). CD4 and FOXP3 positive cells in CNS of the BASY pretreated MOG-immunized mice group significantly reduced from 1.32 to 0.9% and from 3.1 to 1.9%, respectively (Figure 4A,D).

### 3.5. BASY Augmented the Level of Neurotransmitters and Neuronal Hormones in MOG-Immunized Mice Spinal Tissues

**Figure 5:**
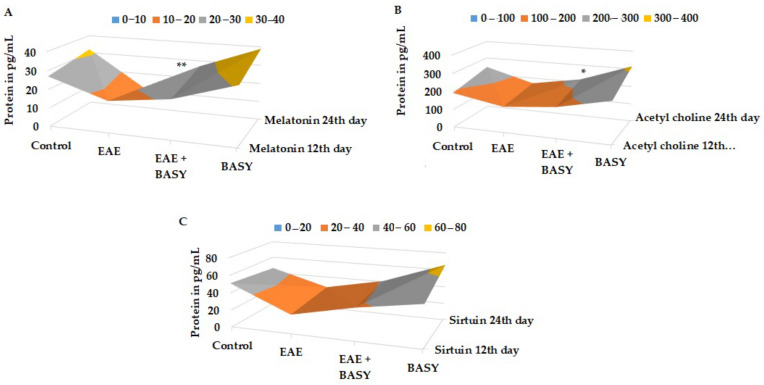
BASY augmented the level of neurotransmitters and neuronal hormones in MOG-immunized mice spinal tissues. (**A**) The peripheral crude lymphocytes were isolated, and total protein was extracted by the rapid freeze method. The melatonin was quantified at two intervals, on the 12th and 24th day of the MOG post-immunization period. The melatonin was quantified using an Abcam hormonal sensitivity kit. (**B**,**C**) Acetylcholine, a substrate of esterase, was quantified using the Sigma-Aldrich ELISA kit. Sirtuin protein was quantified using the Invitrogen Mouse ELISA kit. Data were pooled from three independent experiments and shown as mean ± SD. * *p* < 0.05. ** *p* < 0.01.

The effects of BASY treatment on the levels of neurotransmitters and neuronal hormones in the MOG-induced EAE mice groups are presented in Figure 5. BASY significantly augmented the levels of melatonin on the 12th and 24th day of the MOG post-immunization period in the mice group, as shown in Figure 5A (*p* < 0.05). A slight increase of the acetylcholine and sirtuin levels occurred on the 24th day compared to that on the 12th day of the MOG-immunized mice CNS tissues (Figure 5B,C, respectively).

### 3.6. Gut–Brain Axis Was Regulated by BASY Administration in the MOG-Immunized EAE Mice

**Figure 6:**
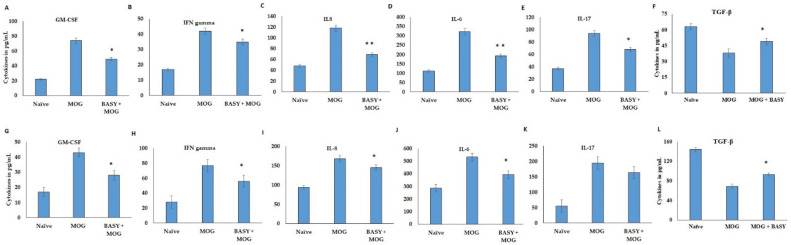
Peripheral and splenic lymphocytes were regulated by BASY administration in MOG- immunized EAE mice. The differential regulation of lymphocytes in MOG-immunized mice was evaluated by progressive inflammatory cytokine markers using an ELISA kit. CD4 and CD65L lymphocytes were isolated from peripheral lymph nodes of MOG-immunized mice and naïve mice. The isolated cells were restimulated with MOG of 1 µg/mL concentration for 18 h and cells were harvested for cytokine quantification. (**A**–**F**) GM-CSF, IFNγ, IL-8, IL-6, IL-17 and TGF-β were quantified in MOG-stimulated peripheral spinal lymph nodes CD4 cells. The isolated cells were re-stimulated with MOG of 1 µg/mL concentration for 12 h and cells were harvested for cytokine quantification. (**G**–**L**) GM-CSF, IFNγ, IL-8, IL-6, IL-17 and TGF-β were quantified in MOG-stimulated splenic CD4 cells. CD4 and CD65L lymphocytes were isolated from splenic lymphoid cells (SLC) of MOG -immunized mice and naïve mice. Data were pooled from three independent experiments and shown as mean ± SD. * *p* < 0.05. ** *p <* 0.01. GM -CSF—granulocyte-macrophage colony-stimulating factor; IFN-γ—interferon gamma; IL—interleukin.

In order to study the effects of the BASY administration on the gut–brain axis, the differential regulation of the inflammatory cytokine markers in lymphocytes of MOG- immunized mice was evaluated and the data presented in Figure 6. The immunized and non-immunized lymphocytes were restimulated with MOG at a concentration of 1 µg/mL. The GM-CSF, IFN γ, IL-8, IL-6 and IL-17 levels were significantly stimulated in MOG-treated CD4 and CD65L cells, in comparison with the BASY-administered MOG mice lymphocytes (Figure 6A). Interestingly, BASY administration in MOG-immunized EAE mice significantly attenuated the GM-CSF, IFN gamma, IL-8, IL-6 and Il-17 (from 72 to 49, from 42 to 35, from 118 to 69, from 324 to 193 and from 94 to 68 pg/mL, respectively), in peripheral lymphocytes (Figure 6A–E); and similarly reduced the cytokine level in the GM-CSF, IFN γ, IL-8, IL-6 and IL-17 (from 43 to 28, from 77 to 56, from 168 to 145, from 533 to 394, and from 195 to 163, respectively) in splenic lymphocytes (Figure 6G–K). The BASY treatment showed significant upregulation of anti-inflammatory mediator TGF-β in both peripheral lymph node CD4 cells and splenic CD lymphocytes (Figure 6F,L).

### 3.7. The Cell Adhesion Molecules Regulated by BASY Treatment in Immunized EAE Mice

**Figure 7:**
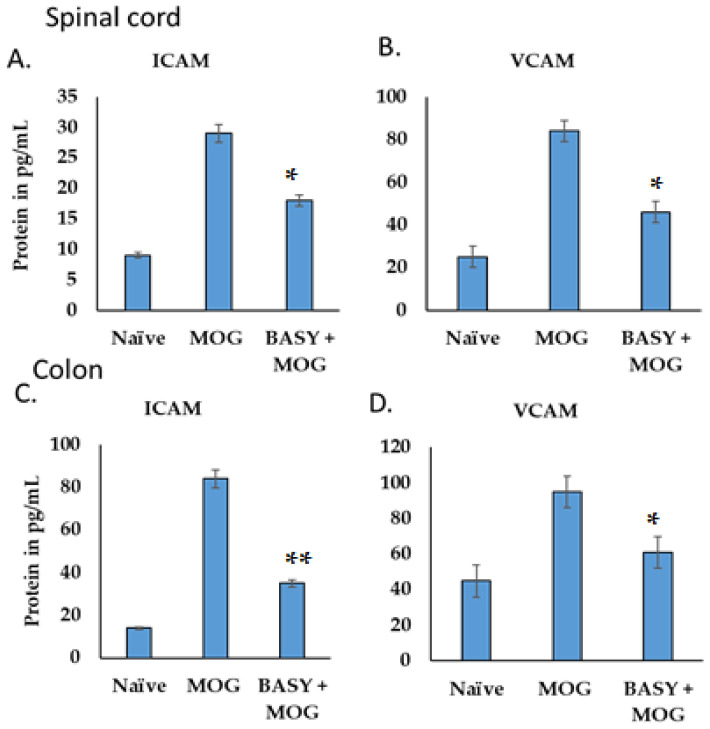
The cell adhesion molecules regulated by BASY treatment in MOG-induced mice. The spinal cord and colon tissues were homogenized and endothelial cell adhesion molecules VCAM and ICAM were quantified using a protein specific ELISA Kit: (**A**,**B**) the differential expression of ICAM and VCAM in the spinal cord after MOG immunization was reduced by BASY treatment; (**C**,**D**) the expression and regulation of the invasion of lymphoid cells from the intestinal lamina propria to the peripheral lymph nodes of the spinal cord was quantified. Data were pooled from three independent experiments and shown as mean ± SD. * *p* < 0.05. ** *p* < 0.01 (n = 5). ICAM—intracellular adhesion molecule-1; VCAM—vascular cell adhesion molecule-1.

To further investigate the regulation pattern of the cell, adhesion molecules (VCAM and ICAM) were estimated in the spinal cord and colon in BASY-administered MOG-immunized EAE mice. The levels of ICAM in both spinal cord and colon tissues were significantly reduced in MOG-immunized EAE mice treated with BASY (Figure 7A,B, respectively: from 29 to 18 and from 84 to 35, respectively; *p* < 0.05) compared with the naïve mice. Similarly, the level of VCAM reduced from 84 to 46 (*p* < 0.01) and from 95 to 61 (*p* < 0.05) in both spinal cord and colon tissues, respectively (Figure 7B,D).

### 3.8. BASY Enhanced the Myelin Protein and Neurotropic Factors in Spinal Cord EAE Mice by Regulating Cell Adhesion Molecules

**Figure 8:**
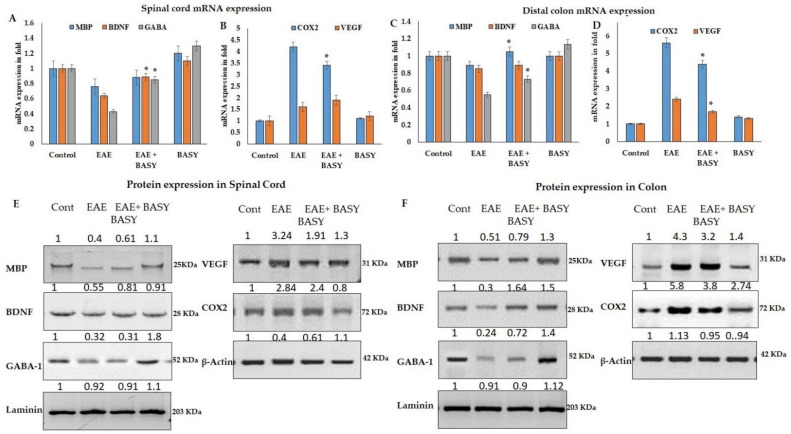
Various markers and neurotropic factors regulated by BASY in MOG-induced EAE mice: (**A**) mRNA expression of MBP, BDNF, and GABA in the spinal cord of MOG-immunized mice. (**B**) mRNA expression of COX-2 and VEGF in the spinal cord of MOG-immunized mice. (**C**) MBP, BDNF, GABA, COX-2, and VEGF protein expression was examined by immunoblot and densitometry analysis in the spinal cord and was normalized with laminin and actin as internal controls. (**D**) mRNA expression of MBP, BDNF, GABA, and COX-2, VEGF in the colon of MOG-immunized mice. (**E**,**F**) MBP, BDNF, GABA, COX-2, and VEGF protein expressions were examined by immunoblot and densitometry analysis in the colon and normalized with actin and laminin as internal controls. Results are presented as mean ± SEM (n = 3). When comparing MOG to MOG+BASY groups, * *p* < 0.05 denotes significance. MBP—myelin basic protein; BDNF—brain-derived neurotrophic factor; GABA—gamma-aminobutyric acid; COX-2—cyclooxygenase-2; and VEGF—vascular endothelial growth factor.

In order to understand the inflammatory marker expression in the histologically-affected spinal cord and colon compared to a relatively non-affected control group, real-time PCR was performed to evaluate the expression level of inflammatory markers in the spinal cord and colon of the MOG-immunized and BASY-treated EAE mice groups (Figure 8). The mRNA expressions of MBP, BDNF and GABA in the spinal cord and colon of MOG-immunized mice significantly increased in the MOG-induced mice groups, whereas BASY treatment reduced the significant level of all markers except MBP and COX-2 compared to the MOG-induced group (*p*< 0.05) (Figure 8B). COX2 expression was comparatively less significant than other tested markers (*p* > 0.0625). Protein expression also reflects the mRNA expression profile, but COX2 showed insignificant changes between the MOG induction and BASY-treated groups (Figure 8C,D). In an overview of the results, BASY in MOG-induced EAE mice enhanced the myelin protein and neurotropic factors in the spinal cord of EAE mice by regulating cell adhesion molecules.

## 4. Discussion

MS is an auto-inflammatory disorder of the CNS that leads to vision impairment and paralysis. Recently, research has focused on microbiota regulating brain functions through the gastrointestinal tract. Several studies have stated that probiotic bacteria positively influence mental health as well as gut health by influencing stress, anxiety and depression, host behavior, and gut-brain signals [46,47,48,49]. MOG was found to induce neuroinflammation or neurotoxicity in mice and has a relation to the gut environment by regulating several factors such as microbiota, inflammatory mediators, immune homeostasis and biochemical markers. The inflammation in GIT is interconnected to the neurons of the limbic system, which induce a stress response in the brain, and the probiotics alleviate paleomammalian cortex and hypothalamic axis activities along with the anti-inflammatory response. The gut–brain axis is a two way communication network of the brain which includes autonomous, central and enteric nervous systems, spinal cord and the hypothalamic pituitary adrenal axis (HPA). The gut–brain axis is interconnected with various factors such as gut or intestinal inflammation and stress which contribute to its impairment. Furthermore, from the gut system, the brain receives the signals which affect the physiology of the intestines [50].

In the current study, the mice were administered BASY for 40 days, and on the 24th day, they were induced with MOG. Next, the pathological signs were observed, such as a flattened tail, paralysis of the hindlimb, and motor sensitivity indicating a disease index during treatment. BASY substantially reduced the disease symptoms and restored the myelin proteins, neurotransmitters and SCFA levels of the gut and neuro environments. These findings indicated that BASY plays an immunomodulatory role. Similar preclinical studies evaluated the use of probiotics as a therapeutic approach against the EAE model, as evidenced by decreasing the hindlimb paralysis, grip study, and pathophysiological symptoms [51]. Therefore, our study validated the gut–brain axis hypothesis through the ameliorative effects of BASY, which could be a potential way to prevent gut–brain-related disorders.

The histopathology analysis revealed that the MOG-induced mice model underwent inflammation, destruction in epithelial and goblet cells, apoptosis and neutrophil infiltration. BASY-preadministration reversed the overall pathological signs in the spinal cord and colon of the mice, rehabilitated gut health, and reversed the histopathological factors in MOG-induced mice. Inflammation and demyelination were significantly reduced in the spinal cord of BASY pre-administered mice. In a previous study, the probiotic *Lactobacillus* sp. significantly lowered the histopathology scores in murine groups [52]. Congruent results were obtained with *Bifidobacterium animalis* [27,51] and other gut-derived microbiota [53] that ameliorated neuroinflammation in EAE mice by reducing infiltration in the myelin sheath.

Cytokines contribute to the inflammation process, and their level is correlated with inflammation severity. The inflammatory cytokines (IL-1β, IL-6, TNF-α, IL-17) were increased significantly in MOG-induced mice; by contrast, in BASY treated mice, they gradually decreased—indicating that BASY displayed an attenuating role on inflammatory cytokines and maintained intestinal integrity via secretion of SCFA and regulatory neurotransmitters in the spinal cord and colon tissue. Several similar studies reported that *E. coli* Nissle strain 1917, *B. animalis*, *L. Plantarum*, *L. johnsonii* and *L. reuteri* decreased the inflammatory cytokines level and inflammation in EAE mice [27,54,55,56]. Moreover, *L. johnsonii* has been reported to secrete mucin to protect the intestinal lining, alleviate anxiety, and anti-inflammatory properties in autoimmune studies [57,58], highlighting that the gut–brain axis is presumably mediated via strain-dependent interactions. Overall, a close correlation exists between oxidative stress and inflammation in the EAE mice model, and MOG stimulates lymphoid dependent MPO expression. In this study, BASY treatment regulated T cell polarization and suppressed MPO levels in EAE mice.

The probiotics secrete SCFA (e.g., propionate, butyrate and caproic acid) during fermentation activities in the intestine and play a key role in the gut–brain cross-talk link [59,60,61,62]. The butyric and caproic acid levels were increased in the BASY treated EAE mice group more than in the other untreated groups. This might be related to the increased production of anti-inflammatory markers and immunomodulatory effects on autoimmune diseases [63]. It has been reported that the administration of *B. subtilis* or *B. licheniformis* increased butyrate production [64,65]. The host lymphoid cell population could interact with probiotic secretory elements to control the induced immunoregulatory effects via inflammatory cells such as T-regulatory cells.

EAE is the immunohistological model of MS aggravated by Th1 and Th17 immune responses. In contrast, Treg cells, which possess the transcription factor FOXP3 and CD4+T cells, play a significant role in mitigating the EAE model through modulating the inflammation process [27]. In our study, BASY showed a substantial improvement in melatonin levels in MOG-immunized mice and a remarkable inhibition of Th1 and Th17 polarization. Comparable results were obtained with *E. coli* Nissle 1917 strain on EAE murine model via a reciprocal manner of the inflammatory cytokines (IL-10) through CD4+T cells in CNS and lymph nodes [56]. Furthermore, a combination between *L. plantarum* A7 and *B. animalis* showed an obvious recovery of the EAE mice model through inhibiting the Th1 and Th17 cells differentiation, and upregulating Foxp3 and GATA3 in the brain and spleen [27]. Such observations indicate a differential expression of lymphoid components in the different microenvironments in MOD-immunized mice.

The T cells and their associated components enhanced the disease signs by secreting proinflammatory cytokines and disturbing cell adhesion molecules [61]. In the current study, the T-lymphocytes from peripheral lymph nodes showed reduced cytokine levels compared to the splenic T-lymphocytes in BASY-administered EAE mice, whereas the expression of cell adhesion molecules such as VCAM and ICAM reduced more significantly in colon tissue than in spinal cord tissue. These results demonstrated that lymph node-expressing cytokines seem to be more sensitive than those in the splenic lymphocytes. The anti-inflammatory markers are reciprocally regulated by BASY treatment in both spinal cord and splenic lymphocytes. These findings are in line with those reported previously by Abadier et al. [61] who confirmed that MOG-induced T cell differentiation is likely to be dependent on endothelial ICAM levels [61]. Additionally, ICAM interactions are responsible for Th17 migration across the brain barrier of the host. Based on these findings, one can speculate that SCFA from probiotics triggered the neurotransmitter via suppression of endothelial ICAM and VCAM markers in the EAE model.

Interestingly, BASY suppressed the demyelination and inflammatory markers in the CNS of EAE mice. Recent evidence showed that proinflammatory cytokines increased the myelin proteins and neurotrophic factors using COX2 and VEGF transcriptional factors in EAE mice models. In the current study, the mRNA and proteins of MBP and BDNF were significantly upregulated in BASY-administered EAE mice, and this was more marked in the spinal cord of than in colon tissue, indicating that probiotic treatment restored the neuronal proteins which are correlated with the levels of neurotransmitters and SCFA. On the other hand, the mRNA and protein of COX2 and VEGF were substantially down-regulated in the EAE mice model, indicating potential host-probiotic interactions take place in the bowel and are transmitted to the neuronal site.

## 5. Conclusions

In this study, BASY increased MOG-induced EAE animals’ body weight and disease index. BASY significantly reduced proinflammatory cytokines (IL1β, IL6, IL8, and IL17), inflammatory progression mRNA, and protein indicators (COX2 and VEGF). SCFA from probiotics suppressed endothelial ICAM and VCAM indicators in the EAE model, triggering the neurotransmitter. BASY therapy increases GABA, decreasing MPO and increasing serotonin, acetylcholine, and sirtuins. EAE mice showed reduced ICAM and VCAM that modulate gut–brain axis neuroinflammation. BASY significantly reduced MS signs and may be employed to develop a probiotic-based diet to increase host gut health and treat inflammatory MS. This work shows that BASY greatly improves neuroprotective characteristics in the MOG-induced murine model, suggesting a new way to prevent MS and other neurological disorders.

## Figures and Tables

**Table 1:** Primer details of the mRNA target.

Primer Name	Forward Primer	Reverse Primer	PCR Product Size in bp
MBP F	: ATTCACCGAGGAGAGGCTGGAA 106 R:	TGTGTGCTTGGAGTCTGTCACC	245
VEGF	AGTCCCATGAAGTGATCAAGTTCA	ATCCGCATGATCTGCATGG	188
GABA-_A_ A1	AAAAGTCGGGGTCTCTCTGAC	CAGTCGGTCCAAAATTCTTGTGA	138
COX2	CAA TTC CCG GAC GTC TAA ACC	CTA GGA CGA TGG GCA TGA AAC	114
BDNF	GCCTTTGGAGCCTCCTCTAC	GCGGCATCCAGGTAATTTT	213
GAPDH	TGGCCTACATGGCCT CCA	TCCCTAGGCCCCTCCTGTTAT	177

**Table 2:** Nutritional content of BEY (~100 mL) in comparison with commercial yogurt.

Nutritional Content	BA 10 × 6 Inoculum	BA 10 × 7.5 Inoculum	BA 10 × 9 Inoculum
Total fat	3.1 * ± 0.2	3.6 ± 0.2	3.9 ± 0.13
Cholesterol	0.038 ± 0.001	0.05 ± 0.01	0.1 ± 0.01
Total carbohydrates	4.4 ± 0.31	3.4 ± 0.2	3.5 ± 0.007
Total protein	3.5 ± 0.01	3.8 ± 0.2	4.1 ± 0.1
Sodium	0.04 ± 0.0031	0.04 ± 0.001	0.04 ± 0.0024
Calcium	0.07 ± 0.28	0.09 ±0.001	0.05 ± 0.002

*: represents a significant value (*p* < 0.05)

## Data Availability

Available from corresponding author upon request.

## References

[1] Morshedi M., Hashemi R., Moazzen S., Sahebkar A., Hosseinifard E.-S. (2019). Immunomodulatory and anti-inflammatory effects of probiotics in multiple sclerosis: A systematic review. J. Neuroinflamm..

[2] Wildner P., Stasiołek M., Matysiak M. (2019). Differential diagnosis of multiple sclerosis and other inflammatory CNS diseases. Mult. Scler. Relat. Disord..

[3] Reich D.S., Lucchinetti C.F., Calabresi P.A. (2018). Multiple Sclerosis. N. Engl. J. Med..

[4] Zeydan B., Kantarci O.H. (2018). Progressive Forms of Multiple Sclerosis: Distinct Entity or Age-Dependent Phenomena. Neurol. Clin..

[5] Lemus H.N., Warrington A.E., Rodriguez M. (2018). Multiple Sclerosis: Mechanisms of Disease and Strategies for Myelin and Axonal Repair. Neurol. Clin.

[6] Khalil M., Teunissen C.E., Otto M., Piehl F., Sormani M.P., Gattringer T., Barro C., Kappos L., Comabella M., Fazekas F. (2018). Neurofilaments as biomarkers in neurological disorders. Nat. Rev. Neurol..

[7] Fisher E., Chang A., Fox R.J., Tkach J.A., Bs T.S., Bs K.N., Rudick R.A., Trapp B.D. (2007). Imaging correlates of axonal swelling in chronic multiple sclerosis brains. Ann. Neurol..

[8] Bielekova B., Martin R. (2004). Development of biomarkers in multiple sclerosis. Brain.

[9] Hashemi R., Morshedi M., Jafarabadi M.A., Altafi D., Hosseini-Asl S.S., Rafie-Arefhosseini S. (2018). Anti-inflammatory effects of dietary vitamin D_3_ in patients with multiple sclerosis. Neurol. Genet..

[10] Brinkmann V. (2009). FTY720 (fingolimod) in Multiple Sclerosis: Therapeutic effects in the immune and the central nervous system. Br. J. Pharmacol..

[11] Ochoa-Repáraz J., Kirby T.O., Kasper L.H. (2018). The Gut Microbiome and Multiple Sclerosis. Cold Spring Harb. Perspect. Med..

[12] Crane J.D., Palanivel R., Mottillo E.P., Bujak A.L., Wang H., Ford R.J., Collins A., Blümer R.M., Fullerton M.D., Yabut J.M. (2015). Inhibiting peripheral serotonin synthesis reduces obesity and metabolic dysfunction by promoting brown adipose tissue thermogenesis. Nat. Med..

[13] Yano J.M., Yu K., Donaldson G.P., Shastri G.G., Ann P., Ma L., Nagler C.R., Ismagilov R.F., Mazmanian S.K., Hsiao E.Y. (2015). Indigenous Bacteria from the Gut Microbiota Regulate Host Serotonin Biosynthesis. Cell.

[14] Ochoa-Repáraz J., Mielcarz D.W., Wang Y., Begum-Haque S., Dasgupta S., Kasper D.L., Kasper L.H. (2010). A polysaccharide from the human commensal Bacteroides fragilis protects against CNS demyelinating disease. Mucosal Immunol..

[15] Wong R.K., Yang C., Song G.-H., Wong J., Ho K.-Y. (2015). Melatonin Regulation as a Possible Mechanism for Probiotic (VSL#3) in Irritable Bowel Syndrome: A Randomized Double-Blinded Placebo Study. Dig. Dis. Sci..

[16] Fan Y., Zhang J. (2019). Dietary Modulation of Intestinal Microbiota: Future Opportunities in Experimental Autoimmune Encepha-lomyelitis and Multiple Sclerosis. Front. Microbiol..

[17] Erny D., Hrabě de Angelis A.L., Jaitin D., Wieghofer P., Staszewski O., David E., Keren-Shaul H., Mahlakoiv T., Jakobshagen K., Buch T. (2015). Host microbiota constantly control maturation and function of microglia in the CNS. Nat. Neurosci..

[18] Freedman S.N., Shahi S.K., Mangalam A.K. (2018). The “Gut Feeling”: Breaking Down the Role of Gut Microbiome in Multiple Sclerosis. Neurotherapeutics.

[19] Kennedy E.A., King K.Y., Baldridge M.T. (2018). Mouse Microbiota Models: Comparing Germ-Free Mice and Antibiotics Treatment as Tools for Modifying Gut Bacteria. Front. Physiol..

[20] Naghavian R., Ghaedi K., Kiani-Esfahani A., Hakemi M.G., Etemadifar M., Nasr-Esfahani M.-H. (2015). miR-141 and miR-200a, Revelation of New Possible Players in Modulation of Th17/Treg Differentiation and Pathogenesis of Multiple Sclerosis. PLoS ONE.

[21] Mousa W.K., Chehadeh F., Husband S. (2022). Microbial dysbiosis in the gut drives systemic autoimmune diseases. Front. Immunol..

[22] Varesi A., Campagnoli L.I.M., Fahmideh F., Pierella E., Romeo M., Ricevuti G., Nicoletta M., Chirumbolo S., Pascale A. (2022). The Interplay between Gut Microbiota and Parkinson’s Disease: Implications on Diagnosis and Treatment. Int. J. Mol. Sci..

[23] Alfonsetti M., Castelli V., D’angelo M. (2022). Are We What We Eat? Impact of Diet on the Gut–Brain Axis in Parkinson’s Disease. Nutrients.

[24] Jiang J., Chu C., Wu C., Wang C., Zhang C., Li T., Zhai Q., Yu L., Tian F., Chen W. (2021). Efficacy of probiotics in multiple sclerosis: A systematic review of preclinical trials and meta-analysis of randomized controlled trials. Food Funct..

[25] Hosseinifard E.-S., Morshedi M., Bavafa-Valenlia K., Saghafi-Asl M. (2019). The novel insight into anti-inflammatory and anxiolytic effects of psychobiotics in diabetic rats: Possible link between gut microbiota and brain regions. Eur. J. Nutr..

[26] Ganji-Arjenaki M., Rafieian-Kopaei M. (2017). Probiotics are a good choice in remission of inflammatory bowel diseases: A meta analysis and systematic review. J. Cell. Physiol..

[27] Salehipour Z., Haghmorad D., Sankian M., Rastin M., Nosratabadi R., Dallal M.M.S., Tabasi N., Khazaee M., Nasiraii L.R., Mahmoudi M. (2017). Bifidobacterium animalis in combination with human origin of Lactobacillus plantarum ameliorate neuroinflammation in experimental model of multiple sclerosis by altering CD4+ T cell subset balance. Biomed. Pharmacother..

[28] Ngo D.-H., Vo T.S. (2019). An Updated Review on Pharmaceutical Properties of Gamma-Aminobutyric Acid. Molecules.

[29] Zheng P., Zeng B., Liu M., Chen J., Pan J., Han Y., Liu Y., Cheng K., Zhou C., Wang H. (2019). The gut microbiome from patients with schizophrenia modulates the glutamate-glutamine-GABA cycle and schizophrenia-relevant behaviors in mice. Sci. Adv..

[30] Cortès-Saladelafont E., Molero-Luis M., Cuadras D., Casado M., Armstrong-Morón J., Yubero D., Montoya J., Artuch R., García-Cazorla À. (2018). Institut De Recerca Sant Joan De Déu Working Group Gamma-aminobutyric acid levels in cerebrospinal fluid in neuropaediatric disorders. Dev. Med. Child Neurol..

[31] Pan S., Wei H., Yuan S., Kong Y., Yang H., Zhang Y., Cui X., Chen W., Liu J., Zhang Y. (2022). Probiotic Pediococcus pentosaceus ameliorates MPTP-induced oxidative stress via regulating the gut microbiota–gut–brain axis. Front. Cell. Infect. Microbiol..

[32] Khalifa A., Sheikh A., Ibrahim H.I.M. (2022). *Bacillus amyloliquefaciens* Enriched Camel Milk Attenuated Colitis Symptoms in Mice Model. Nutrients.

[33] Arab H.H., Salama S.A., Eid A.H., Omar H.A., Arafa E.-S.A., Maghrabi I.A. (2014). Camel’s milk ameliorates TNBS-induced colitis in rats via downregulation of inflammatory cytokines and oxidative stress. Food Chem. Toxicol..

[34] Hailu Y., Hansen E.B., Seifu E., Eshetu M., Ipsen R., Kappeler S. (2016). Functional and technological properties of camel milk proteins: A review. J. Dairy Res..

[35] Lei Q., Wu T., Wu J., Hu X., Guan Y., Wang Y., Yan J., Shi G. (2021). Roles of A synuclein in Gastrointestinal Microbiome Dysbiosis related Parkinson’s Disease Progression (Review). Mol. Med. Rep..

[36] El-Sayed M., Awad S. (2019). Milk Bioactive Peptides: Antioxidant, Antimicrobial and Anti-Diabetic Activities. Adv. Biochem..

[37] Al-Dhabi N.A., Arasu M.V., Vijayaraghavan P., Esmail G.A., Duraipandiyan V., Kim Y.O., Kim H., Kim H.-J. (2020). Probiotic and Antioxidant Potential of *Lactobacillus reuteri*LR12 and *Lactobacillus lactis*LL10 Isolated from Pineapple Puree and Quality Analysis of Pineapple-Flavored Goat Milk Yoghurt during Storage. Microorganisms.

[38] Jäger A., Dardalhon V., Sobel R.A., Bettelli E., Kuchroo V.K. (2009). Th1, Th17, and Th9 Effector Cells Induce Experimental Autoimmune Encephalomyelitis with Different Pathological Phenotypes. J. Immunol..

[39] Peters A., Pitcher L.A., Sullivan J.M., Mitsdoerffer M., Acton S.E., Franz B., Wucherpfennig K., Turley S., Carroll M.C., Sobel R.A. (2011). Th17 Cells Induce Ectopic Lymphoid Follicles in Central Nervous System Tissue Inflammation. Immunity.

[40] Zaiss M.M., Rapin A., Lebon L., Dubey L.K., Mosconi I., Sarter K., Piersigilli A., Menin L., Walker A.W., Rougemont J. (2015). The Intestinal Microbiota Contributes to the Ability of Helminths to Modulate Allergic Inflammation. Immunity.

[41] Pennartz S., Reiss S., Biloune R., Hasselmann D., Bosio A. (2009). Generation of Single-Cell Suspensions from Mouse Neural Tissue. J. Vis. Exp..

[42] De Bondt M., Hellings N., Opdenakker G., Struyf S. (2020). Neutrophils: Underestimated Players in the Pathogenesis of Multiple Sclerosis (MS). Int. J. Mol. Sci..

[43] Carriel V., Campos A., Alaminos M., Raimondo S., Geuna S. (2017). Staining Methods for Normal and Regenerative Myelin in the Nervous System. Histochemistry of Single Molecules.

[44] Mirzaei R., Bouzari B., Hosseini-Fard S.R., Mazaheri M., Ahmadyousefi Y., Abdi M., Jalalifar S., Karimitabar Z., Teimoori A., Keyvani H. (2021). Role of microbiota-derived short-chain fatty acids in nervous system disorders. Biomed. Pharmacother..

[45] Kaliyamoorthy V., Jacop J.P., Thirugnanasambantham K., Ibrahim H.I.M., Kandhasamy S. (2022). The synergic impact of lignin and Lactobacillus plantarum on DSS-induced colitis model via regulating CD44 and miR 199a alliance. World J. Microbiol. Biotechnol..

[46] Mu C., Yang Y., Zhu W. (2016). Gut Microbiota: The Brain Peacekeeper. Front. Microbiol..

[47] Foster J.A., Neufeld K.-A.M. (2013). Gut–brain axis: How the microbiome influences anxiety and depression. Trends Neurosci..

[48] Vuong H.E., Yano J.M., Fung T.C., Hsiao E.Y. (2017). The Microbiome and Host Behavior. Annu. Rev. Neurosci..

[49] Sarkar A., Lehto S.M., Harty S., Dinan T.G., Cryan J.F., Burnet P.W.J. (2016). Psychobiotics and the Manipulation of Bacteria–Gut–Brain Signals. Trends Neurosci..

[50] Enck P., Aziz Q., Barbara G., Farmer A.D., Fukudo S., Mayer E.A., Niesler B., Quigley E.M.M., Rajilic-Stojanovic M., Schemann M. (2016). Irritable bowel syndrome. Nat. Rev. Dis. Prim..

[51] Calvo-Barreiro L., Eixarch H., Ponce-Alonso M., Castillo M., Lebrón-Galán R., Mestre L., Guaza C., Clemente D., del Campo R., Montalban X. (2020). A Commercial Probiotic Induces Tolerogenic and Reduces Pathogenic Responses in Experimental Autoimmune Encephalomyelitis. Cells.

[52] Sengul N., Tore F., Isik S., Aslim B., Ucar G., Firat T., Ciftci S.Y., Kukner A. (2022). Effects of Probiotic Bacteria on Central Neuronal Activation in Experimental Colitis. Turk. J. Gastroenterol..

[53] Mangalam A., Shahi S.K., Luckey D., Karau M., Marietta E., Luo N., Choung R.S., Ju J., Sompallae R., Gibson-Corley K. (2017). Human Gut-Derived Commensal Bacteria Suppress CNS Inflammatory and Demyelinating Disease. Cell Rep..

[54] Mu Q., Tavella V.J., Luo X.M. (2018). Role of Lactobacillus reuteri in Human Health and Diseases. Front. Microbiol..

[55] Xin J., Zeng D., Wang H., Sun N., Zhao Y., Dan Y., Pan K., Jing B., Ni X. (2019). Probiotic Lactobacillus johnsonii BS15 Promotes Growth Performance, Intestinal Immunity, and Gut Microbiota in Piglets. Probiotics Antimicrob. Proteins.

[56] Secher T., Kassem S., Benamar M., Bernard I., Boury M., Barreau F., Oswald E., Saoudi A. (2017). Oral Administration of the Probiotic Strain Escherichia coli Nissle 1917 Reduces Susceptibility to Neuroinflammation and Repairs Experimental Autoimmune Encephalomyelitis-Induced Intestinal Barrier Dysfunction. Front. Immunol..

[57] Davoren M.J., Liu J., Castellanos J., Rodríguez-Malavé N.I., Schiestl R.H. (2018). A novel probiotic, Lactobacillus johnsonii 456, resists acid and can persist in the human gut beyond the initial ingestion period. Gut Microbes.

[58] Jang H.-M., Lee K.-E., Lee H.-J., Kim D.-H. (2018). Immobilization stress-induced Escherichia coli causes anxiety by inducing NF-κB activation through gut microbiota disturbance. Sci. Rep..

[59] Pascale A., Marchesi N., Marelli C., Coppola A., Luzi L., Govoni S., Giustina A., Gazzaruso C. (2018). Microbiota and metabolic diseases. Endocrine.

[60] Silva Y.P., Bernardi A., Frozza R.L. (2020). The Role of Short-Chain Fatty Acids From Gut Microbiota in Gut-Brain Communication. Front. Endocrinol..

[61] Liu Y., Alookaran J.J., Rhoads J.M. (2018). Probiotics in Autoimmune and Inflammatory Disorders. Nutrients.

[62] He J., Guo K., Chen Q., Wang Y. (2022). Jirimutu Camel milk modulates the gut microbiota and has anti-inflammatory effects in a mouse model of colitis. J. Dairy Sci..

[63] Luu M., Monning H., Visekruna A. (2020). Exploring the Molecular Mechanisms Underlying the Protective Effects of Microbial SCFAs on Intestinal Tolerance and Food Allergy. Front. Immunol..

[64] Xu Y., Yu Y., Shen Y., Li Q., Lan J., Wu Y., Zhang R., Cao G., Yang C. (2021). Effects of Bacillus subtilis and Bacillus licheniformis on growth performance, immunity, short chain fatty acid production, antioxidant capacity, and cecal microflora in broilers. Poult. Sci..

[65] Musa B.B., Duan Y., Khawar H., Sun Q., Ren Z., Mohamed M.A.E., Abbasi I.H.R., Yang X. (2019). *Bacillus subtilis*B21 and *Bacillus licheniformis*B26 improve intestinal health and performance of broiler chickens with *Clostridium perfringens*-induced necrotic enteritis. J. Anim. Physiol. Anim. Nutr..

